# Factors Affecting Intention among Students to Be Vaccinated against A/H1N1 Influenza: A Health Belief Model Approach

**DOI:** 10.4061/2011/353207

**Published:** 2011-12-20

**Authors:** Sharon Teitler-Regev, Shosh Shahrabani, Uri Benzion

**Affiliations:** ^1^Department of Economics and Management, The Max Stern Academic College of Emek Yezreel, Emek Yezreel 19300, Israel; ^2^Department of Economics, The Western Galilee College, P.O. Box 2125, Akko 24121, Israel

## Abstract

The outbreak of A/H1N1 influenza (henceforth, swine flu) in 2009 was characterized mainly by morbidity rates among young people. This study examined the factors affecting the intention to be vaccinated against the swine flu among students in Israel. Questionnaires were distributed in December 2009 among 387 students at higher-education institutions. The research questionnaire included sociodemographic characteristics and Health Belief Model principles. The results show that the factors positively affecting the intention to take the swine flu vaccine were past experience with seasonal flu shot and three HBM categories: higher levels of perceived susceptibility for catching the illness, perceived seriousness of illness, and lower levels of barriers. We conclude that offering the vaccine at workplaces may raise the intention to take the vaccine among young people in Israel.

## 1. Introduction

On June 11, 2009, the World Health Organization issued a statement declaring that the A/H1N1 influenza virus (henceforth, the swine flu) had reached pandemic proportions [1, 2]. Around 30,000 people worldwide have died because of this virus, including 17,000 in the USA and around 90 in Israel [3, 4].

Governments geared up to launch national programs to vaccinate the population against the swine flu. Most governments planned to vaccinate groups of people at risk and healthcare personnel in the first stage and the entire population in the second stage [5]. Yet, while compliance rates were quite high in some countries, such as Australia (67%) and France (60%) [1, 6], in others the rates remained quite low. For example, according to a representative survey carried out by the CDC in 2010, by February 2010 only 23.4% of Americans had been vaccinated against the swine flu, while in Israel by February 2010 less than 10% had taken the new vaccine [7]. 

The purpose of the current study was to examine the factors affecting the intention among students in Israel to be vaccinated against the swine influenza. We chose to focus on this group of young people because the swine flu affected not only at-risk groups but also young people in the labor force. In fact, a major difference between seasonal influenza A and the 2009 outbreak of H1N1 influenza was the age distribution of life-threatening cases and deaths [8]. According to media reports, most deaths from H1N1 2009 influenza were among young and middle-aged adults. In contrast, most deaths from seasonal influenza A occur in the older population, while deaths of young people due to seasonal influenza are rare [9]. Indeed, the true public health burden of H1N1 2009 influenza may be better measured by the number of deaths rather than by the number of life-years lost [8].

Several recent studies have examined willingness to be vaccinated against the swine flu in developed countries. For example, the results of Blendon et al. [10] showed that among a representative sample of the US population just 40% of adults were “absolutely certain” they would get the H1N1 vaccine for themselves. In addition, those who were not “absolutely certain” they would get the H1N1 vaccine cited the following as the “major” reasons for their thinking: (1) concerned about side effects of the vaccine, (2) they are not at risk of getting a serious case of the illness, and (3) there are alternative medications to treat H1N1. 

Similar results were found for the general population in France [11, 12]. Raude et al. [11] indicate the following reasons for vaccine acceptance among French adults: (1) beliefs associated with severity and personal vulnerability to the illness, (2) perception of vaccine efficacy and safety, and (3) trust/distrust of those advocating the vaccine. In addition, the results of Setbon and Raude [12] indicated that level of worry, risk perception, and previous experience with vaccine against seasonal flu in France consistently predicted swine flu vaccination. Similar results were also cited as predictors for swine flu vaccination acceptance in Australia [6]. Yet, 67% of the population in Australia was willing to be vaccinated against the swine flu [6]. Finally, in Israel, a telephone survey conducted several months after the peak of the outbreak indicated low compliance for the A/H1N1 vaccine (17%) [13]. The results showed that apathy, fear, and distrust were motives leading to noncompliance. 

Willingness to get vaccinated against a given infectious illness is recognized as a major issue affecting the success of vaccination programs. For example, according to the Theory of Reasoned Action [14], which has explained and predicted a variety of human behaviors, the most important determinant of *behavior *is a person's behavior *intention* [15]. In addition, several studies have shown that the intention-behavior correlation is quite strong. For example, the meta-analysis study by Armitage and Conner [16] showed this correlation as *r* = .47, Randall and Wolf [17] reported a corresponding relationship of  .45 (98 studies), and Sheeran and Orbell [18] reported a mean correlation of  .44 (28 studies of condom use). 

Our study examines the factors affecting the intention to get vaccinated against the swine influenza using the Health Belief Model (HBM) [19] as a conceptual framework. The HBM, which has largely been tested empirically, explains and predicts preventive health behavior in terms of belief patterns, focusing on the relationship between health behaviors and utilization of health services. According to the HBM, getting vaccinated against influenza depends on the following predictors: perceived susceptibility to influenza, beliefs about severity of influenza, perceived benefits of the vaccine in preventing influenza, and perceived barriers to getting vaccinated [20–23]. Based on these findings with respect to seasonal influenza, we expect to find similar predictors for the intention to get vaccinated against swine flu.

The current study was conducted in December 2009 in Israel, after several people in the country died of the swine flu and after the topic had received extensive media coverage. In addition, the timing of the survey was one month after the Israeli government launched a vaccination program offering the vaccine to people at risk and to healthcare personnel and before the vaccine was offered to everyone free of charge. Nevertheless, the government announced that in the second stage the vaccine would be offered to everyone free of charge.

The study contributes to the existing literature by

- examining the factors affecting the intention to get vaccinated against the swine influenza among students in Israel;
- examining the *conditional* intentions to get vaccinated;
- comparing sociodemographic characteristics of the intention to get vaccinated against the swine flu to characteristics marking the intention to get vaccinated against the seasonal flu.

## 2. Methods

### 2.1. Sample

The study's sample included 387 students studying in various academic departments at four institutions of higher education in Israel: Technion, Max Stern Academic College of Emek Yezreel, Western Galilee College and Kinneret College on the Sea of Galilee. 

### 2.2. Design and Procedure

Over a period of three weeks in December 2009, after several people had died from the swine flu in Israel and in other countries, the questionnaires were randomly distributed among the classes. A short verbal explanation of the purpose of the study was given prior to distributing the self-administered questionnaire. In addition, we explained that participation in the study was voluntary and provided details about the researchers. The questionnaire was distributed during class and collected after about 30 minutes. Students could choose not to fill in the questionnaire. The study was approved by the Ethics Committee of the Max Stern Academic College of Emek Yezreel.

### 2.3. Measures

The research questionnaire was partially based on the questionnaire developed by Blue and Valley, [21] and its Hebrew version [23]. The final version of the questionnaire was decided upon after analyzing data from a pilot questionnaire distributed at one academic institute. 

The questionnaire consisted of the following parts: (1) items requesting sociodemographic information, including age, marital status, education, nationality, and membership in a particular Health Maintenance Organization (henceforth, HMO); (2) questions concerning vaccination against seasonal influenza and the respondent's intentions to get vaccinated against the seasonal influenza and the swine flu in the next 12 months, on a 5-point scale ranging from 1 (“certainly I will get the vaccine in the next year”) to 5 (“I will definitely not get the vaccine in the next year”); (3) reasons for intending to get or not get vaccinated against the swine flu, including knowledge about the vaccine (“There is not enough knowledge about the side effects of vaccine”) and evaluation of risk of contracting seasonal flu and swine flu with and without vaccination; (4) items measuring HBM variables, including the four categories of susceptibility, seriousness, benefits, and barriers, as well as the categorical variables of perceived infection risk and health motivation (see Table 8 in the appendix). This part of the questionnaire was based on the Blue and Valley [21] tested and retested questionnaire and its Hebrew version, which was validated in the study by Shahrabani and Benzion [23]. In addition, the final versions of the HBM scales were decided upon after we distributed a pilot questionnaire at one of the institutions. Finally, only those scales whose internal consistency reliability (Cronbach's alpha) was higher than 0.60 were retained. Items in the HBM predictor categories and the categorical variables were measured on a 5-point Likert-type scale, with the following possible responses: strongly agree (1), agree (2), neither agree nor disagree (3), disagree (4), and strongly disagree (5). The items in the *barriers* category were reverse scored, so that, like the other categories, lower scores indicated lower levels. The scores on each of the scales were averaged to form the independent variables. The values of the independent variable predicted the participant's intention to get the swine flu vaccination. 

### 2.4. Data Analysis

The statistical package SPSS 16 was used for statistical analysis of the data. Chi-square tests were used to determine how selected categorical variables, including demographic factors (e.g., gender), were related to the dependent variable of intention to get vaccinated in the coming year. To facilitate easier and more instructive interpretation, we carried out a binary logistic regression (and not ordinal). Therefore, we transformed the initial 5-point Likert scale of intention to get vaccinated into a binary scale: the dependent variable was a dichotomous variable that is equal to one if an individual said that he/she “definitely intends” or “probably intends” to get a flu shot in the next year, and zero for “definitely do not intend” and “probably do not intend.” The answer “do not know” was excluded. 

The statistical significance of the difference between the means of the continuous variable (e.g., age, summary scales, etc.) for two different groups (e.g., those who intend to take the vaccine *versus* those who have no intention to get the vaccine) was determined by *t*-test. Multiple logistic regressions were conducted to identify the impact of demographic factors, factors derived from the HBM model, and other factors of interest regarding intention to get vaccinated in 2009.

## 3. Results

### 3.1. Descriptive Statistics

Of the 450 participants who were given the questionnaires, 422 (93.7% response rate) returned a usable questionnaire. The major reason cited by those who chose not to participate in the survey was that they did not have the time to fill in the questionnaire at the end of the class and during the break between classes. In addition, 35 questionnaires (out of 422) were completed by individuals over the age of 40 and were excluded from the sample. Therefore, the final sample included 387 participants. This sample size provided a power of 80% and more for the main outcome (intention to get the swine flu vaccination) for factors with OR 1.5 and more.

Table 1 summarizes the basic demographic information and characteristics for the sample. The table reveals that the sample included 38% men and 61% women (about 1% missing values) under the age of 40, with an average age of 25.9. In addition, 87% of the respondents were Jews and the rest (13%) belonged to other religious groups. Moreover, 81% were born in Israel and 19% were new immigrants (who immigrated to Israel after 1990).  Table 7 in the appendix shows the characteristics for the samples for each of the four institutions.

We also found that in December 2009, only 13.9% of the participants (51 out of 387) indicated they intended to be vaccinated against the swine flu. However, among the 34.5% of the sample who had experience with seasonal flu shots over the last five years, more than 16% said that they intended to take the new vaccine against the swine flu. 

In addition, we found that the average perceived risk of swine flu is significantly lower than the average perceived risk of catching the seasonal flu (40.5% versus 50.6%, resp., *P* value < 0.001). Nevertheless, we did not find any significant difference between the percentage of those intending to take the swine flu shot (13.9%) and the percentage of those intending to take the seasonal flu shot (12%).

Table 2 summarizes the characteristics for the sample according to intention to get vaccinated against the swine flu and against the seasonal flu. In this sample we omitted the answers of respondents who said they “do not know” whether or not to take the vaccine. The table reveals that 21.7% of the men and 17.5% of the women (*P* value = 0.4) said they intended to take the swine flu shot, while only 13.7% of the men and 13.4% of the women (*P* value = 0.54) said they intended to take the seasonal flu shot. 

Another result revealed by the table is that those who perceived a high risk of contracting the swine flu were more willing to take the vaccine, compared to those who perceived the risk as medium or low (37.9%, 19.2%, and 11.2%, resp., *P* value = 0.004). For the perceived risk of contracting the seasonal flu, the results were similar (22.9%, 15.6%, and 7.4%, resp., *P* value = 0.03).

### 3.2. Main Reasons for Intention to Accept or Refuse the Swine Flu Shot

Table 3 summarizes the main reasons indicated for the intention to accept (Table 3(a)) or to refuse (Table 3(b)) the swine flu shot during the next year. Respondents could select more than one reason. In addition, 29% of the respondents (114 out of 387) gave reasons both for intending to take the vaccine and for intending not to take it. The results in Table 3(a) show that the top motivators for the intention to get the swine flu shot in 2010 were (a) to reduce my chances of contracting influenza (59%); (b) because I have a chronic illness (41%); (c) so as not to transfer the illness to other people (33%). 

Table 3(b) shows the following main reasons for the intention to refuse the swine flu vaccine in 2010: (a) not enough knowledge about the safety of the vaccine and its side effects (66%); (b) the vaccine is not effective (27%); (c) the vaccine is not good for one's health (25%); 

### 3.3. Effect of Offering Vaccination at Workplace on Intention to Get Vaccinated 

Table 4 shows participants' intentions to get vaccinated in the coming year if the vaccine were to be offered at their workplace or at the institution where they are studying. According to Table 4, 11.4% of the 210 students who declared they do not intend to get vaccinated during the next 12 months and 33.3% of those who were not sure about their intention to get vaccinated indicated that if the vaccine were to be offered at their place of work or study, they would get vaccinated during the coming year. In other words, these results indicate that for the entire sample, the incentive to get vaccinated substantially increases from 13.9% to 26.7% when the vaccine is available at places of work or study. 

### 3.4. Results of the HBM Categories

Table 5 shows the HBM model categories mean values and the categorical variables (defined in Table 8 in the appendix) mean values as indices on a 5-point Likert scale measured by intention to get the swine flu shot in 2010. The scale ranged from “strongly agree”-1, to “strongly disagree”-5. The Cronbach's alpha coefficients for the HBM categories were perceived susceptibility (HBM1) 0.69, perceived seriousness (HBM2), perceived benefits (HBM3) 0.744, perceived barriers (HBM4) 0.641, and health motivation 0.678 (For the seriousness category, we used only the question “the swine flu can be a serious disease one can die from” since the other questions in the same category resulted in a relatively low Cronbach's alpha coefficient.).

As expected, the results in Table 5 indicate that on average those who intend to get the swine flu shot perceived the swine flu to be a more serious illness than did those who did not intend to take the vaccine. In addition, the first group felt they were more susceptible to illness, perceived higher risk of infection, perceived more benefits from vaccination, and had fewer barriers to getting the swine flu shot than did the second group. Moreover, on average the individuals who intend to get the vaccination had higher levels of health motivation than those who did not intend to be vaccinated. 

### 3.5. Results of the Analytical Model 

The analytical model examines the effect of each of the explanatory variables on the dependent variable when controlling for all other variables including the sociodemographic characteristics.  Table 6 shows the results of the logistic model regressions. In Table 6, the dependent variable is a dichotomous variable that is equal to one if an individual “definitely intends” or “probably intends” to get a swine flu shot in the next year and to zero for a response of “definitely do not intend” and “probably do not intend.”

The independent variables are: gender (male or female), perceived risk of infection without vaccination, knowledge about the vaccine, number of vaccinations against the seasonal flu in the past five years, and the HBM categories and health motivation. 

The results in Table 6 show that the significant factors *positively* affecting the intention to get the swine flu shot in the next year include higher perceived risk of catching swine flu without vaccination (OR = 0.47, *P* value = 0.04) and three HBM categories: higher levels of perceived susceptibility of illness (OR = 0.23, *P* value = 0.01), higher levels of perceived seriousness of illness (OR = 0.18, *P* value = 0.01) and lower levels of barriers (OR = 0.32, *P* value = 0.01). In addition, the results indicate that previous experience with seasonal flu vaccination (the number of flu shots in the last five years) increases the intention to get the swine flu shot (OR = 1.75, *P* value = 0.04). 

## 4. Summary and Discussion

Previous studies investigating the determinants of vaccine acceptance during the 2009 (H1N1) pandemic influenza have focused on target groups such as healthcare workers or the general population [12, 13, 24]. The current study examines the factors affecting intention to get vaccinated against the swine flu among students in Israel, since the H1N1 pandemic influenza affected not only at-risk populations but also young people. 

Vaccination may prevent illness and reduce direct medical costs and productivity costs due to absence from work or from institutions of higher education (relevant especially for students). Yet, in December 2009, despite extensive media coverage given to the deaths of young people from the H1N1 influenza, we found that only 13.9% of the students in the sample said they intend to get the vaccine against the swine flu. 

Our results indicate that the top motivator for intention to get the swine flu shot among this young age group was “to reduce the chances of contracting influenza.” This reason was also mentioned as the major motivator for intention to get the seasonal flu shot [23]. The major reason for intention to refuse the swine flu vaccine was: “I do not have enough knowledge about the vaccine's safety and its side effects” (similar to the findings with respect to the general population in Greece [25]). Yet, this reason is different from the major reason mentioned for rejecting the seasonal flu shot: “There are many strains of influenza” [23]. Therefore, it may be that with respect to the swine flu, these results suggest that the public wanted more information about swine flu and the vaccine against it before being vaccinated since they are apprehensive regarding a new vaccine about which there is little safety/efficacy data.

Our analysis of the HBM categories showed that on average individuals who intend to be vaccinated perceived swine influenza as a more serious illness and felt they were more susceptible to it than did those who do not intend to be vaccinated. This result is compatible with findings with respect to the general population in France [11]. In addition, we found that individuals who intend to get the swine flu shot perceived more benefits from vaccination and had fewer barriers to getting the swine flu shot than did those that did not intend to get the vaccination. Moreover, on average those who expressed their intention to be vaccinated had higher levels of health motivation than those who did not intend to be vaccinated. In general, these results are compatible with previous studies regarding seasonal influenza [21, 22]. 

When we controlled for all other variables by using the analytical model, we found that higher perceived risk of contracting the swine flu and higher number of vaccinations against seasonal flu in the past five years significantly increase the intention to take the swine flu shot among the students. These results are in line with the findings of previous studies with respect to the general population [12, 25]. Actually, it may be that those who have had good experiences with seasonal flu shots in the past (e.g., perceived that the vaccine prevented illness) will be less afraid to try the new vaccine against the swine flu. 

Our results also indicate that the odds of intending to be vaccinated increase significantly with higher level of perceived seriousness of the illness, higher level of perceived susceptibility to the illness, and lower levels of barriers. It is interesting that the other HBM category, perceived benefits of the vaccine, which was found to be a significant factor explaining intention to take the seasonal flu shot [23], was not found to be a significant factor in explaining the intention to take the swine flu shot. 

In light of our results, we can conclude that concerns about safety and the possibility of side effects need to be addressed. In addition, our results suggest that offering the vaccine free of charge at workplaces and at institutions of higher education may increase the intention to take the vaccine (from 13.9% to 26.7%). Shahrabani and Benzion [23] found that offering vaccinations at workplaces in Israel significantly affects the intention to get the seasonal flu shot as well as the actual acceptance of vaccination. Vaccination at workplaces may reduce the indirect costs of vaccination (including inconvenience), and might also give people more confidence about the vaccine and its safety.

Finally, the study's limitations must be acknowledged when interpreting the results. The study sample was limited to four higher education institutions. For this reason it is not certain that the findings can be generalized to students in other institutions or even to all students in these four institutions. For example, only 13% of the sample comprised non-Jews, though the proportion of non-Jews at these institutions is higher. Another point to consider is that the percentages of women in the samples of two out of the four institutions were higher than the overall percentages of women at those institutions. Yet, we did not find any significant differences between men and women in their intention to get the swine flu vaccine. Furthermore, in the logistic regression gender was not a significant factor affecting intention to get vaccinated. Another limitation of the study was the fact that the survey was conducted in December 2009, two months after the outbreak of the swine flu in Israel. However, at that point the vaccine was available only to people at risk and to healthcare personnel and had not yet been offered to the general population. Future research could possibly examine the intention to get the vaccine before the influenza season begins and compare it to the actual acceptance of the vaccine at the end of the season.

## Figures and Tables

**Table 1:** Sample's characteristics.

	Number	(%)
Gender		
Male	146	37.7
Female	236	61
Missing values	5	1.3
Age group		
Under 25	240	62
25–30	102	26.4
31–40	45	11.6
Marital status		
Married	63	16.3
Unmarried	310	80.1
Missing values	4	1.0
Religion		
Jews	337	87.1
Others	50	12.9
Immigrants or born in Israel		
Immigrant	82	21.2
Israel	305	78.8

**Table 2:** Comparison of sample characteristics: Intention to get the swine flu vaccination versus intention to get the seasonal flu vaccination.

		Intention to get swine flu vaccination				Intention to get seasonal flu vaccination			
		Number	No (%)	Yes (%)	*P*-Value	Number	No (%)	Yes (%)	*P*-Value
Gender	Male	106	78.3	21.7	0.40	117	86.3	13.7	0.54
	Female	154	82.5	17.5		186	86.6	13.4	
Age group	<25	107	80.4	19.6	0.64	144	85.4	14.6	0.79
	25–30	125	82.4	17.6		68	84.7	10.3	
	31–40	32	75.0	25.0		28	82.0	18.0	
Marital status	Married	45	75.6	24.4	0.40	52	80.8	19.2	0.18
	Unmarried	219	81.7	18.3		256	86.7	13.3	
Religion	Jews	228	82.9	17.1	0.02	267	86.9	13.1	0.10
	Others	36	66.7	33.3		41	78.0	22.0	
Immigrants or born in Israel	Immigrant	57	82.5	17.5	0.70	68	83.8	16.2	0.37
	Israel	207	80.2	19.8		240	86.2	13.8	
Five-year seasonal flu shot status	never	46	87.0	13.0	0.23	52	90.4	9.6	0.20
	1 and above	218	79.4	20.6		256	84.8	15.2	
Perceived risk of	High	29	62.1	37.9	0.004	35	77.1	22.9	0.03
contracting influenza	Medium	104	80.8	19.2		128	84.4	15.6	
without vaccine*	Low	98	88.8	11.2		108	92.6	7.4	

*For the intention to get the swine flu vaccination (columns 3–6), the perceived risk refers to the swine flu, while for intention to get the seasonal flu vaccination (columns 7–10) the perceived risk refers to the seasonal flu.

**Table:** (a) Reasons for intention to get the swine flu shot (*N* = 183)

Reasons for getting flu shot*	Number of respondents selecting response	% of respondents selecting response
To reduce my chances of contracting swine flu	108	59
I have a chronic illness	75	41
Not to transfer the illness to other people	60	33
The swine flu shot was free of charge	21	11
I do not want to miss any work because of swine flu	16	9

**Table:** (b) Reasons for intention to reject the swine flu shot* (*N* = 303)

Reasons for rejecting flu shot*	Number of respondents selecting response	% of respondents selecting response
Not enough knowledge about the vaccine safety	199	66
The vaccine is not effective	81	27
The vaccine is not good for health	76	25
Do not like injections	67	22
Potential side effect	67	22
I do not suffer from chronic illness	66	22
There are better ways	63	21

*Respondents could select more than one reason.

**Table 4:** Intention to get vaccinated if vaccine is offered at place of work or study.

		Intend to get vaccinated if vaccine		
		is offered at place of work or study		
		Yes *n* (%)	No *n* (%)	Do not know *n* (%)
Intention to get vaccinated in the next 12 months	No	24 (11.4)	129 (61.4)	57 (27.2)
	Do not know	33 (33.3)	11 (11.1)	55 (55.5)
	Yes	39 (78.0)	6 (11.8)	5 (10.2)

**Table 5:** Mean values of Health Belief Model (HBM) measures by intention to get the swine flu shot in the next year.

Scale*	Intend to get vaccinated		Do not intend to get vaccinated		*t* test (*P* value)
	*N*	Mean (SD)	*N*	Mean (SD)	
Susceptibility	51	2.08 (0.7)	212	3.01 (0.86)	8.02 (0.00)
Seriousness	50	1.60 (0.76)	212	2.36 (1.11)	−5.8 (0.00)
Benefits	50	2.54 (0.821)	211	3.33 (0.77)	−6.2 (0.00)
Barriers	44	3.42 (0.9)	200	3.12 (1.01)	1.98 (0.05)
Health Motivation	51	2.56 (0.87)	207	2.83 (0.81)	−2.02 (0.05)
Perceived infection risk	50	3.58 (0.93)	210	4.1 (0.87)	−3.16 (0.00)

*The 5-point scale “strongly agree” (1) to “strongly disagree” (5).

**Table 6:** Results of logistic regression for dependent variables: intention to get vaccinated.

Dependent variable			
Explanatory variables	Intention to get vaccinated (*N* = 285, Pseudo *R* ^2^ = 0.475)		
	Odds ratio	Std. err.	*P* > |*z*|

Gender (base = male)	4.9	0.9	0.09
Perceived infection risk**	0.47	0.37	**0.04**
HBM1-Susceptibility**	0.23	0.015	**0.01**
HBM2-Seriousness**	0.18	0.65	**0.01**
HBM3-Benefits**	0.7	0.6	0.43
HBM4-Barriers^+^	0.32	0.47	**0.01**
Health motivation**	1.3	0.45	0.55
Number of flu shots in the last 5 years	1.75	0.27	**0.04**

**The 5-point scale ranged from “strongly agree” (1) to “strongly disagree” (5).

^+^HBM4 was reversed score.

**Table 7:** Characteristics of the sample by institution.

	Kinneret College		Emek Yezreel College		Western Galilee College		Technion	
	Number	(%)	Number	(%)	Number	(%)	Number	(%)
Gender								
Male	47	47.5	57	29.1	32	48.5	10	38.5
Female	50	50.5	138	70.4	33	50	15	57.7
Missing value	2	2	1	0.5	1	1.5	1	3.8
Religion								
Jews	79	79.8	176	89.8	58	87.9	24	92.3
Others	20	20.2	20	10.2	8	12.1	2	7.7

Average age	Years		Years		Years		Years	
	27.3		24.5		28.4		25.0	

**Table 8:** HBM categories and categorical variables.*

Variables	Statements
HBM categories	
Susceptibility	Working with many people each day increases my chances of getting the swine flu
	I worry a lot about getting the swine flu
Seriousness	The swine flu can be a serious disease one can die from
Benefits	Getting the swine flu shot will prevent me from getting the flu
	Getting the swine flu shot will prevent me from missing work
	I would not be afraid of getting the swine flu if I got the swine flu shot
Barriers	Getting the swine flu shot can be painful
	Getting the swine flu shot is time consuming
	There are too many risks in getting the swine flu shot
	I am concerned about having a bad reaction to the swine flu shot
	People often get sick from flu injections

Categorical variables	
Perceived infection risk	My chances of getting the swine flu are good
Health motivation	I eat a well-balanced diet
	I follow medical orders because I believe they will benefit my state of health
	I search for new information related to my health

*The 5-point scale for the categories ranged from “strongly agree” (1) to “strongly disagree”.

## Appendix

For more details see Tables 7 and 8.
